# Exploring Active Peptides with Antimicrobial Activity In Planta against *Xylella fastidiosa*

**DOI:** 10.3390/biology11111685

**Published:** 2022-11-21

**Authors:** Kaoutar El Handi, Miloud Sabri, Franco Valentini, Angelo De Stradis, El Hassan Achbani, Majida Hafidi, Maroun El Moujabber, Toufic Elbeaino

**Affiliations:** 1 Laboratory of Plant Biotechnology and Valorization of Bio-Resources, Faculty of Sciences, Moulay Ismail University, Meknes 11201, Morocco; 2 Laboratory of Phyto-Bacteriology and Biocontrol, Plant Protection Unit-National Institute of Agronomic Research INRA, Meknes 50000, Morocco; 3 Istituto Agronomico Mediterraneo di Bari (CIHEAM-IAMB), Via Ceglie 9, 70010 Valenzano, BA, Italy; 4 Productions Végétales, Animales et Agro-Industrie, Faculté des Sciences, Ibn Tofail University, Kenitra 14000, Morocco; 5 National Research Council of Italy (CNR), Institute for Sustainable Plant Protection (IPSP), University of Bari, Via Amendola 165/A, 70126 Bari, BA, Italy

**Keywords:** bactericidal, v-qPCR, fluorescence microscopy, transmission electron microscopy, in-vivo and in-vitro assays

## Abstract

**Simple Summary:**

*Xylella fastidiosa* is one of the most harmful plant bacteria in the world, which could lead to significant economic losses. The lack of direct and efficient therapies to treat plants infected with this bacterium has involved researchers in the search for new approaches and compounds capable of countering this alarming pathogen. Antimicrobial peptides (AMPs) are one of the promising candidates for sustainable and eco-friendly treatment for agricultural applications. Accordingly, nine AMPs against *Xf* were evaluated, three of which were shown to be promising in limiting *Xf* growth and its biofilm formation in in vivo and in vitro experiments, and thus, are considered as emerging biocontrol agents against *Xf* infection.

**Abstract:**

*Xylella fastidiosa (Xf)* is a xylem-limited quarantine plant bacterium and one of the most harmful agricultural pathogens across the world. Despite significant research efforts, neither a direct treatment nor an efficient strategy has yet been developed for combatting Xylella-associated diseases. Antimicrobial peptides (AMPs) have been gaining interest as a promising sustainable tool to control pathogens due to their unique mechanism of action, broad spectrum of activity, and low environmental impact. In this study, we disclose the bioactivity of nine AMPs reported in the literature to be efficient against human and plant pathogen bacteria, i.e., *Escherichia coli*, *Staphylococcus aureus*, and *Pseudomonas aeruginosa*, against *Xf,* through in vitro and in vivo experiments. Based on viable-quantitative PCR (v-qPCR), fluorescence microscopy (FM), optical density (OD), and transmission electron microscopy (TEM) assays, peptides Ascaphin-8 (GF19), DASamP1 (FF13), and DASamP2 (IL14) demonstrated the highest bactericidal and antibiofilm activities and were more efficient than the peptide PB178 (KL29), reported as one of the most potent AMPs against *Xf* at present. Furthermore, these AMPs showed low to no toxicity when tested on eukaryotic cells. In in planta tests, no *Xf* disease symptoms were noticed in *Nicotiana tabacum* plants treated with the AMPs 40 days post inoculation. This study highlighted the high antagonistic activity of newly tested AMP candidates against *Xf*, which could lead to the development of promising eco-friendly management of *Xf*-related diseases.

## 1. Introduction

*Xylella fastidiosa* (*Xf*) is a fastidious bacterium that infects more than 664 different plants including landscape trees, wild and ornamental plants, and crop species [1]. Several valuable crops have suffered serious economic losses caused by *Xf* epidemics [2,3]. The earliest field outbreak of *Xf* in the European Union (EU) occured in October 2013, when the existence of this quarantined phytopathogen in the olive trees of Apulia in southern Italy was reported by the Italian Phytosanitary Authorities [4]. The *Xf* disease, i.e., olive quick decline syndrome (OQDS), known to lead to extreme leaf scorch and branch dieback, poses one of the most harmful threats to landscape agriculture in Europe [5,6]. *Xylella fastidiosa* subspecies *pauca* strain De Donno was isolated in 2014 from OQDS-stricken olive trees and its complete genome has been sequenced [7,8]. The crucial pathogenic mechanism of *Xf* depends on biofilm formation causing the obstruction of xylem vessels [9,10]. Within the biofilm, the cells organize their function and behavior to persist in their environment and to survive. Currently, many measures are adopted (such as application of N-acetyl-L-cysteine (NAC), menadione, and copper (II) sulfate, etc. [11]) to manage and monitor *Xf*-associated diseases, aiming to stop and limit the spread of the bacterium. However, no strategy was able to completely cure plants infected by *Xf*. Nowadays, environmentally friendly options and sustainable strategies that conform to European natural trends are still needed. In this regard, antimicrobial peptides (AMPs) have always been promising candidates and an optimistic sustainable tool owing to their low toxicity to the host plants and their low propensity to incite bacteria-acquired resistance [12].

Additionally, AMPs are characterized as natural polypeptide arrangements, composed of hydrophobic and cationic amino acids (2 to 50) with antimicrobial activity and are well-known for being a host defense peptide [13]. They are an element of the innate immunity of plants and animals [14]. Based on amino acid sequences, AMPs are structurally classified into five distinct subgroups, relying on their amino acid sequences, protein structure, and net charge: cationic α-helical AMPs, anionic AMPs, extended cationic AMPs, β-sheet AMPs, and antimicrobial protein fragments [15]. AMPs are categorized into two models with respect to their action mechanism: the non-pore models (e.g., the carpet model) and the transmembrane pore model, which includes both toroidal pore and barrel-stave pore [15]. They interact with bacterial membranes by binding to their cationic residues, which initially attract negatively charged components of the lipidic outer membrane by electrostatic attraction. Some peptides can penetrate bacterial cells and further interact with nucleic acids and other intracellular targets [16]. 

Due to the difficulties of working with *Xf*, studies on the inhibition by AMPs have received little attention in the past. Nonetheless, studies have reported the minimal inhibitory concentration (MIC) of cecropin/magainin derivatives and cecropins A and B [17], gomosin [18], indolicidin and dermaseptin derivatives [19], and radicinin [20]. BP178 peptide is a synthetic BP100-magainin derivative possessing strong inhibitory activity against plant pathogenic bacteria. In the case of *Xf*, BP178 is the only antibacterial agent that has been tested in vitro and demonstrated high performance against *Xf* strains [21]. Its lytic activity against *Xf* cells was identified as the main mode of action, with pore formation and disorganization of the cell membrane [22]. Previous researchers have emphasized the bactericidal and/or bacteriostatic action of several peptides with antibiofilm activity against other Gram-negative and Gram-positive bacteria, namely *Erwinia amylovora*, *Pseudomonas aeruginosa*, *Escherichia coli*, *Klebsiella pneumoniae*, and *Staphylococcus aureus*, or sequences with both antibiofilm and antibacterial activity [23,24,25]; these peptides might be considered good candidates to be tested against *Xf*.

Thus, the aims of the present work were (i) to disclose for the first time the bioactivity of nine AMPs against *Xf*, previously reported in the literature for their bactericidal and/or bacteriostatic action and with antibiofilm activity against other Gram-negative and Gram-positive bacteria; (ii) to study their dose–effect relationships; (iii) to analyze their antibiofilm activity; (iv) to study the cell damage induced by AMPs at the ultrastructural level; and (v) to investigate their possible toxicity and in planta antagonistic effects. 

## 2. Materials and Methods

### 2.1. Bacterial Strains and Media

*Xanthomonas albilineans* (*Xa*), strain CFBP2523 (GenBank accession number, MDCB00000000), was purchased from the “French Collection of Plant-associated Bacteria” (Angers, France) and was grown on LPGA for 2 days at 28 °C [26]. *Xylella fastidiosa* subsp. *pauca*, strain De Donno (GenBanK accession number, NZ_CM003178), was cultured in BCYE (buffered charcoal yeast extract) [27], and then colonies were scraped from the agar surface after growing for 10 to 15 days at 28 °C. Cells were suspended in sterile succinate citrate phosphate (SCP) buffer to achieve a suspension of an OD (optical density) at 600 nm of 0.32, corresponding to 10^8^ CFU/mL. 

### 2.2. Peptide Synthesis

The AMPs adopted in our screening were chosen based on their efficient antimicrobial activity reported in the literature against human and plant bacteria, i.e., *Escherichia coli*, *Staphylococcus aureus*, and *Pseudomonas aeruginosa* (Table 1). Peptides were synthesized by ProteoGenix (Schiltigheim, France) and their purity (>95%) was assessed by high-performance liquid chromatography (HPLC). Lyophilized peptides were dissolved in sterile distilled water to a stock concentration of 1 mM, and a 0.22 μm pore size filter was used for sterilization. 

### 2.3. Antibiogram Assays

Due to the particular nature of *Xf,* and to establish an approximate starting concentration of peptides with which *Xf* cells could be afterward challenged, prior screening was performed using the surrogate *Xa*, a phylogenetically close and relatively swift-growing organism [26].

Six different peptide concentrations (150, 100, 50, 25, 12, and 6 µM) were tested and 5 µL from the 6 concentrations of each AMP were applied directly on *Xa* cells cultured on LPGA medium. Effects of AMPs on *Xa*, i.e., inhibition halos, were checked after 48 h of incubation at 25 °C, using the Gel Doc XR+ (Bio-Rad Laboratories, Hercules, CA, USA). Results were considered positive (+) when halo formation occurred, and negative (−) when there was no observable halo.

For *Xf*, bacteria and AMPs were co-plated in the following order: Three drops of *Xf* suspension (10^8^ CFU/mL), each containing 30 µL, were placed at the top of the petri dish, 1 cm apart, and allowed to slowly flow down to the opposite side of the plate, resulting in three parallel rows of *Xf*. After drying under the laminar flow hood, 5 μL of 50, 25, and 12 μM of AMPs were placed on top of each row, and PBS was used as a negative control. The growth inhibition zones were checked after 10–15 days post incubation at 28 °C and evaluated as the distance between the top of the plate and the edges of *Xf* growth. Three independent replicates were performed for both bacteria.

### 2.4. Effect of Selected AMPs on Xylella fastidiosa Growth

Effects of AMPs on *Xf* growth were assessed by contact test assays, coupled with OD600 measurements over 24 h. *Xf* suspension (180 µL, 10^8^ CFU/mL) in PD2 (Pierce disease 2 media) was mixed with 20 µL of the corresponding peptide dilutions (50, 25, and 12 µM). Bacterial growth was measured by monitoring the OD600 using the NanoDrop™ One/OneC Microvolume UV-Vis Spectrophotometer (ThermoFisher Scientific, Waltham, MA, USA), after 0 min, 0.5 h, 1.5 h, 3 h, 18 h, and 24 h of incubation at 28 °C. Experiments were performed two times, with three replicates for each treatment.

### 2.5. Viable-Quantitative PCR (v-qPCR) and Fluorescence Microscopy (FM)

The bactericidal activity of the AMPs was assessed by contact test, coupled with viable-quantitative PCR (v-qPCR) [30]. AMP-treated *Xf* cells, together with heated *Xf* cells (95 °C for 10 min) used as positive controls, were subject to v-qPCR assay using PMAxx™ (Biotium, Rome, Italy), which is a photo-reactive dye that binds with high affinity to DNA templates. Upon peptide lysis activity, the PMAxx™ dye becomes covalently attached to disrupted DNA that cannot be amplified by PCR. PMAxx™ dye is designed to interact with cell membrane permeability; in a population of live and dead cells, only dead cells are susceptible to DNA modification due to compromised cell membranes. This unique feature makes PMAxx™ highly useful in selective detection of live bacteria by qPCR. Therefore, dilutions of a homogeneous cell suspension (from 10^8^ to 10^2^ CFU/mL) of viable or dead cells to a total volume of 200 µL in DNA low-binding tubes were prepared. Samples were kept in the dark for 8 min at room temperature (25 °C), following a 15-min photoactivation with PMA-LiteTM LED Photolysis Device, at a final concentration of 7.5 µM. The DNA of *Xf* was extracted using the CTAB protocol [30] and was analyzed in duplicate by a TaqMan-based qPCR assay using the target-specific primers *Xf*-F: 5′-CACGGCTGGTAACGGAAGA-3′ and *Xf*-R: 5′-GGGTTGCGTGGTGAAATCAAG-3′ and the probe *Xf*-Prb: 5′ 6FAM-TCGCATCCCGTGGCTCAGTCC-BHQ-1-3′ [30]. Three replicates for each dilution and AMP experiment were performed.

The reduction in viability, expressed as log10 CFU/mL, was obtained by interpolating the CT value from each sample against the respective standard curve for each strain and subtracting it from the non-treated control (Log10 (N0/N). Results were analyzed with a one-way analysis of variance (ANOVA) followed by the post-hoc Student-Newman-Keuls test. SPSS statistical package software (SPSS for Windows, Version 20, SPSS Inc., Chicago, IL, USA) was used for the statistical analysis of data.

For the fluorescence microscopy (FM), aliquots of *Xf* suspension were incubated with AMPs at 50 μM for 1 h at room temperature. The untreated control reaction consisted of a bacterial suspension with only sterile distilled water. The LIVE/DEAD ^®^BacLight™ (Molecular Probes) viability kit was used to assess the viability of bacteria cells treated with peptides. The kit contains two nucleic acid dyes SYTO 9 and propidium iodide (PI) that allow distinguishing live cells with intact plasma membranes (green channel) from dead bacteria with compromised membranes by the peptide’s activity (red channel). Photomicrographs were taken with a Nikon E800 microscope using fluorescein isothiocyanate (480/30 excitation filter, DM505 dichroic mirror, 535/40 emission filter) and tetramethyl rhodamine isocyanate (546/10 excitation filter, DM575 dichroic mirror, 590 emission filter) fluorescence filter sets.

### 2.6. Transmission Electron Microscopy (TEM) of AMPs against Xylella fastidiosa

*Xylella fastidiosa* cells in suspension (10^8^ CFU/mL) were incubated with peptides at 50 μM for 1 h at room temperature. NAC-treated *Xf* suspensions were used as control reactions for the lytic activity. Preparations were observed under TEM (FEI MORGAGNI 282D, Hillsboro, OR, USA) by the dip method; i.e., the carbon-coated copper/rhodium grids were incubated with treated and untreated bacterial suspension for 5 min and afterward were rinsed with 200 μL of distilled water. Negative staining was obtained by floating the grids on 200 μL of 0.5 % *w/v* UA-zero EM STAIN (Agar-Scientific Ltd., Stansted, UK) solution and was observed under the EM using an accelerating voltage of 80 kV.

### 2.7. Antibiofilm and Planktonic Cell Activity

The effects of AMPs on *Xf* biofilm formation and planktonic cells were further investigated. NAC, which has been reported to reduce biofilm formation of *Xf*, was used at 25 and 50 µM as a positive control reaction. *Xf* suspension (180 µL) in PD2 was mixed with 20 µL of the corresponding peptide dilution in 96-well plates. Microplates were incubated for 20 days at 28 °C under constant shaking (140 rpm). Afterward, planktonic cells were recovered from the media and transferred into a new microplate, and their OD600 was measured.

To assess the biofilm formation, the original 96-well plate was rinsed 3 times carefully with sterile distilled water, stained with 250 µL of crystal violet (0.1%) for 20 min, and rinsed with sterile distilled water 3 times to discard excess dye. Eventually, with 250 µL of a mixture of ethanol/acetone (4:6) for 10 min, crystal violet adhered to the biofilm was solubilized and the OD595 was measured. DNA extraction was carried out for each sample and analyzed in duplicate by a TaqMan-based qPCR. For dose–response modelling of biofilm formation, the percentage of biofilm (B) was calculated according to the following formula: B = (Oi/Oc) × 100, where Oi is the OD595 of the treatment and Oc is the OD595 of the untreated control [21].

### 2.8. Toxicity Evaluation of AMPs

The eventual toxicity of the AMPs was evaluated on plant (*Nicotiana tabacum* cv. Xanthi) and animal (horse) eukaryotic cells. Using a syringe, 100 µL of peptide solutions (50 and 25 μM) were injected into fully expanded tobacco leaves. Three infiltrations per plant were performed, for a total of six replicates. The negative control reaction consisted of using sterile distilled water instead of AMPs. Plants were maintained in a glasshouse at 25 °C for 48 h. The possible toxicity on leaves was determined by measuring the diameter of lesions caused by the AMPs.

Erythrocytes from a horse blood sample were provided by the University of Bari, Department of Veterinary Medicine (Valenzano, Italy). The erythrocytes were challenged with AMPs at a final concentration of 50 µM. The hemolytic activity was observed under a light microscope.

### 2.9. In Planta Antagonistic Activity of AMPs against Xylella fastidiosa

To evaluate the antagonistic activity of AMPs that could interfere with the *Xf* virulence inside the plant, *Xf* infection and AMP application in the stem were carried out in *Nicotiana tabacum (*var. *Xanthi*) plants, maintained in a quarantine laboratory. Ten groups of six plants each were mechanically syringe-injected with 100 μL (10^7^ CFU/mL) of *Xf* subsp. *pauca* isolate De Donno [21]. Immediately after the drops dried, 100 μL of AMPs (at concentrations of 50 and 25 μM) was administered at the same points using sterile distilled water as mock. Two groups of six plants were only injected with peptides at the two different concentrations. Leaf lesions, a characteristic symptom of tobacco plants infected with *Xf*, were inspected for 60 days post inoculation (dpi). Two independent experiments were conducted. TaqMan-based qPCR assays were carried out to detect the presence of AMP-treated *Xf* infection in the leaves above the inoculation point. Briefly, 1 g of leaf tissue was ground with 2 mL of CTAB buffer [30] and heated at 65 °C for 30 min. The plant extract was centrifuged at 13,000× *g* for 15 min, and the supernatant was twice washed with chloroform and precipitated in cold isopropyl alcohol. The pellet (total DNA) was eluted in 100 μL of sterile water, whilst 50 ng of DNA was used in qPCR assays.

## 3. Results

### 3.1. Screening of Potent AMP Activities against Xanthomonas albilineans and Xylella fastidiosa

**Figure 1:**
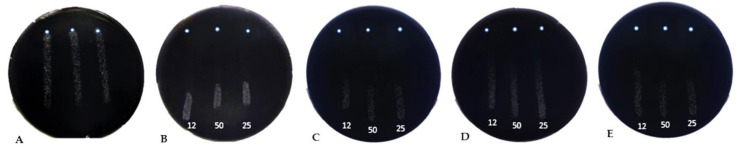
Antibiogram assays of AMPs used against *Xylella fastidiosa.* (**A**) Untreated *Xf* used as a negative control; (**B**) GF19-treated *Xf*; (**C**) FF13-treated *Xf*; (**D**) KL29-treated *Xf*; and (**E**) IL14-treated *Xf*. In petri dishes (**B**–**E**), AMP drop locations are indicated by white dots at the top of the plate. AMP concentrations (μM) are shown at the bottom of the plates.

In this study, nine selected peptides were evaluated for their antimicrobial activities against the surrogate *Xa* as part of an initial in vitro screening. Of note, *Xa* and *Xf* show two different growths (in time and shape) on petri dishes and their antibiograms resulting from the AMP challenge are somehow incomparable. However, the results showed that all peptides were active against *Xa*, with MICs ranging from 6 μM to 50 μM (Table 2). This result provides an information about which concentrations and AMPs can be utilized against *Xf*. In addition, all AMPs were able to reduce the growth of *Xf* at varying degrees at 50, 25, and 12 μM. Three AMPs (GF19, FF13, and IL14) were particularly highly active and were able to strongly curtail *Xf* growth. FF13 and IL14 showed high antimicrobial activity, causing very large inhibition zones (Figure 1), with mean growth zones of 15.6 and 13.7 mm, respectively. GF19 exhibited distinct inhibitory activity, whereas KL29, which is currently considered to be the most potent peptide against *Xf*, showed a clearing zone of 11.8 mm, representing lower activity than the three AMPs newly tested here.

### 3.2. Optical Density of AMP-Treated Xylella fastidiosa Cells

**Figure 2:**
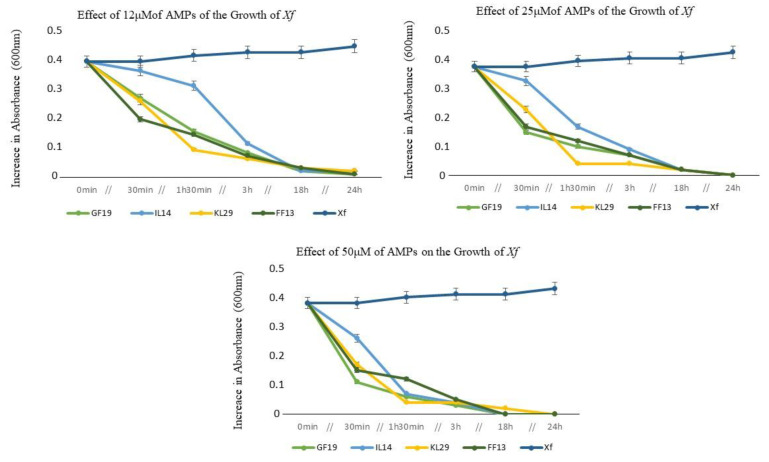
Optical density values showing the AMPs’ inhibitory activity against *Xf* cells, at different concentrations. The curves show means of three replicates per treatment.

The dose–effect reactions of *Xf* to the various AMPs were evaluated over 24 h of incubation. In general, all AMPs at different concentrations (12, 25, and 50 μM) showed reductive activity against *Xf* cells within the first 30 min of contact, and were still particularly highly functional up to 3 h post contact (Figure 2). Furthermore, this reductive activity persisted for 24 h post contact, until the total exhaustion of *Xf* cells, where GF19, FF13, and IL14 each showed the greatest activity at 50 μM. At 24 h of incubation and at 12 µM or higher concentration, all peptides showed significant cell reduction activity (up to 99% of reduction) (Figure 2).

### 3.3. Effect of AMPs on Xylella fastidiosa: V-qPCR, Fluorescence, and Transmission Electron Microscopy

**Figure 3:**
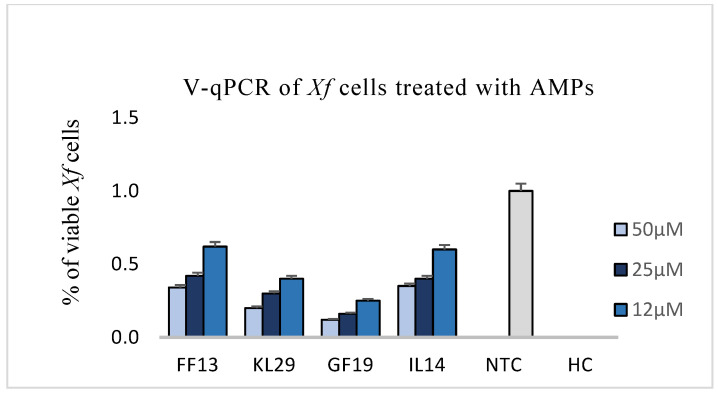
Viability of AMP-treated *Xylella fastidiosa* cells at 12, 25, and 50 µM. NTC and HC are untreated and heated *Xf* cells, respectively.

The reduction in viability of AMP-treated *Xf* was evaluated at 12, 25, and 50 µM. The PMAxx™ assay, used for testing the viability of bacteria and consequently only intercepts dead bacteria with compromised cell membranes, showed that GF19 was highly active at 25 and 50 µM concentrations, leading to a more-than-fourfold reduction in viability compared to untreated *Xf* cells (Figure 3).

**Figure 4:**
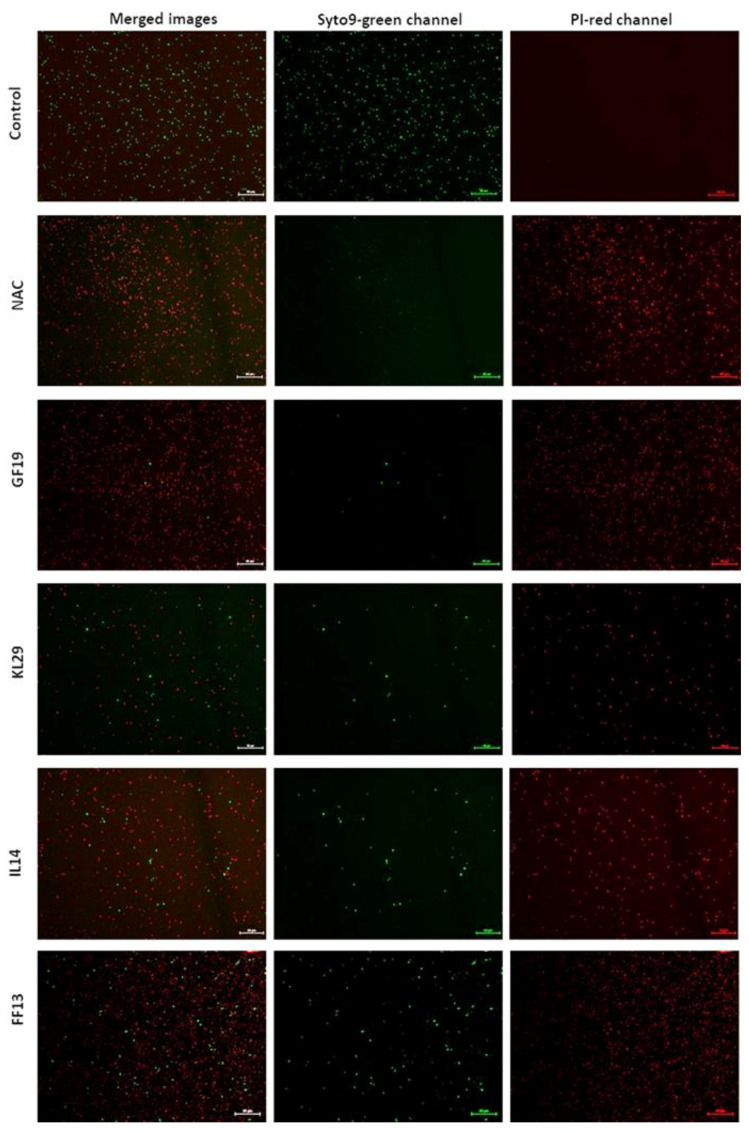
Fluorescent micrographs showing AMP-treated *Xylella fastidiosa* cells at 50 µM. Green and red fluorescence represent viable and dead cells, respectively. NAC—N-acetyl-L-cysteine-treated *Xf* cells. Bar: 50 µm.

In addition, FF13, IL14, and KL29 exhibited high activity, with a twofold reduction in cell viability. The AMPs’ activity against *Xf* cells was also examined under FM using two distinct dyes able to distinguish between live cells with intact plasma membranes (green channel) and dead bacteria with membranes compromised by the peptide activity (red channel). The examined micrographs of all tested peptides showed that GF19 and KL29 had the highest lytic activities, inducing intense red channels indicating the huge number of dead cells. However, the IL14 and FF13 micrographs showed a discreet green fluorescence, highlighting a moderate lytic activity of these peptides against *Xf* (Figure 4).

**Figure 5:**
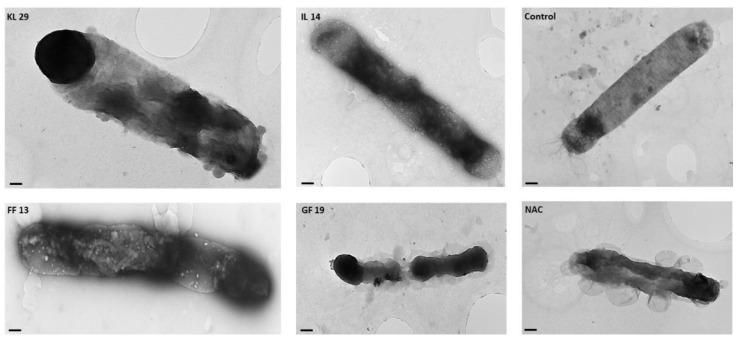
Transmission electron micrographs of AMP-treated *Xf* cells (IL14, GF19, KL29, FF13), showing structural, cell wall, and cytoplasm alterations. Control: untreated *Xf* cells. NAC—N-acetyl-L-cysteine-treated *Xf* cells. Bar: 100 nm.

Under EM, micrographs of all AMP-treated *Xf* cells showed different morphological changes at the structural and cell wall membrane levels. GF19-treated *Xf* cells had damaged and fragmented cell walls, with complete destruction of the outer and inner bacterial membranes (Figure 5). IL14- and FF13-treated *Xf* cells showed cytoplasmic condensation, loss of interior appearance, and alteration of the outer membrane. KL29- and NAC (a bactericidal compound used as a control)-treated cells showed pore and protrusion formation and condensed cytoplasmic outflow from the outer membrane (Figure 5).

### 3.4. Effects of AMPs against Xylella fastidiosa Biofilm Formation

**Figure 6:**
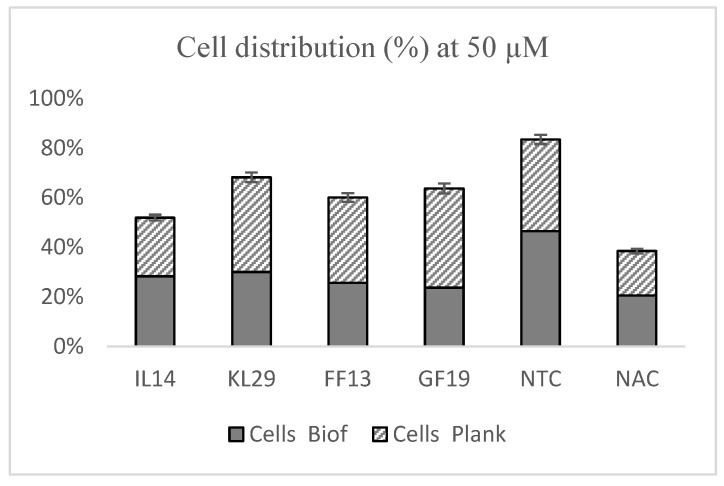
Effect of AMPs on *Xf* biofilm formation. NTC—untreated *Xf* cells. NAC—N-acetyl-L-cysteine-treated *Xf* cells.

*Xf* biofilm formation was evaluated upon exposure to 50 μM of GF19, FF13, IL14, reference peptide KL29, and NAC. The results show that all tested peptides were able to reduce biofilm formation by 40% to 50% compared to the NTC (Figure 6). GF19 interfered with biofilm formation in the same manner as NAC. GF19, FF13, and IL14 each showed greater performance than KL29, which is notoriously known to have high antagonistic activity against *Xf* biofilm formation. Furthermore, these peptides conditioned the ratios of planktonic cells within a range of 13.8% (Figure 6).

### 3.5. Evaluation of AMPs’ Toxicity on Eukaryotic Cells

**Figure 7:**
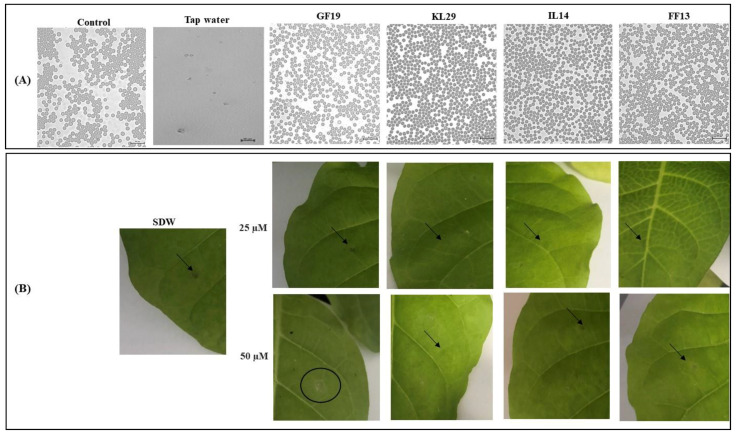
Micrographs showing (**A**) intact horse erythrocyte membranes and (**B**) non-lysed tobacco leaves challenged with different concentrations of AMPs for exploring their possible cytotoxicity. Control: untreated erythrocytes. Tap water-treated erythrocytes and sterile distilled water (SDW) injection in tobacco leaves were used as positive controls. Arrows show the AMP injection sites. A circle shows small leaf necrosis as a response to light hypersensitivity from GF19 at 50 μM. Bar: 20 µm.

The toxicity of AMPs was explored on model cells of animal (horse erythrocytes) and plant (tobacco leaves), for possible future application of these AMPs as eco-friendly candidates in the control of *Xf*. The reason for using such a heterologous system (horse erythrocytes) was to investigate whether these AMPs can differentiate between animal and plant cells regarding their possible toxicities, and to ensure that they do not pose any danger to users. At 25 and 50 μM, all AMPs induced neither a hypersensitivity reaction in the tobacco leaves nor morphological abnormalities in the cellular structures of the erythrocytes (Figure 7). However, at 50 μM, GF19 generated a light hypersensitive reaction in tobacco leaves, i.e., halo-shaped tissue dissolving at 7 mm in diameter (data not shown). These findings were taken as prospective proof for their possible safe use; however, further assessments are needed to scrutinize their possible wide application in different hosts and environments.

### 3.6. AMPs’ Efficiency in the Control of Xylella fastidiosa In Planta

**Figure 8:**
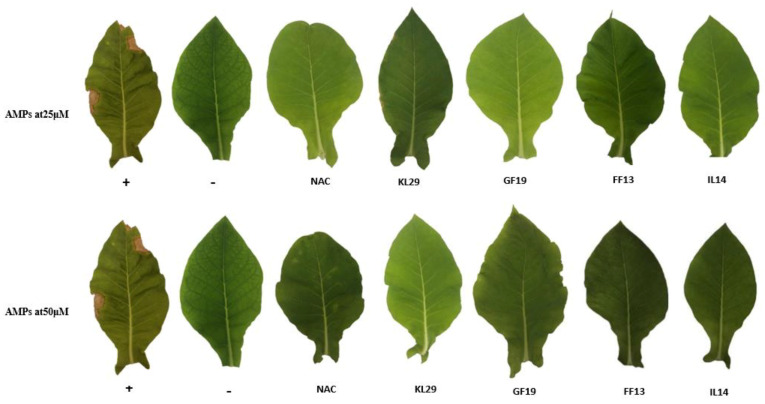
Tobacco leaves showing the interaction between AMPs and *Xf* at 45 dpi. +: *Xf*-infected tobacco leaf showing scorch symptoms at 30 dpi; -: untreated tobacco leaf showing no symptoms. NAC—N-acetyl-L-cysteine-treated *Xf*-infected leaf showing no symptoms, used as a positive control treatment.

**Figure 9:**
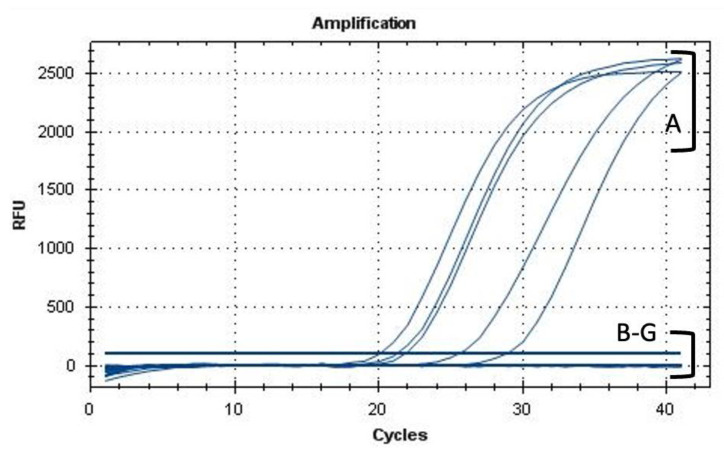
qPCR assay showing DNA amplification curves obtained from (A) *Xf-*infected tobacco plants untreated with AMPs (positive controls); *Xf-*infected tobacco plants treated with (B) NAC, (C) KL29, (D) GF19, (E) FF13, and (F) IL14; and (G) uninfected tobacco leaves used as a negative control.

To contrast the *Xf* disease infection in planta, the efficiency of the AMPs was evaluated in *Xf*-infected tobacco plants. At 30 dpi, the untreated *Xf*-infected tobacco plants showed marginal and apical scorched areas on leaves, whereas AMP-treated *Xf*-infected tobacco plants did not develop any symptoms, similarly to those treated with N-acetyl-L-cysteine, known for its bactericidal activity against *Xf* (Figure 8). AMP-treated *Xf*-infected tobacco plants maintained a healthy appearance until 50 dpi. However, at 25 µM and 45 dpi, KL29-treated *Xf*-infected tobacco plants developed light leaf scorch symptoms that were less intense than those in the untreated *Xf*-infected tobacco plants (Figure 8). These in planta results, i.e., presence and absence of *Xf* infection and symptoms, were confirmed by qPCR assays conducted on different leaves situated above the inoculation point (Figure 9).

## 4. Discussion and Conclusions

The extreme difficulties of managing *Xf* infections have led to the search for new bactericides able to treat the disease with an eco-friendly approach without compromising the contrasting efficiencies. Many active compounds including toxins, phenolic acids, antibiotics, and AMPs have been reported to be effective against numerous *Xf* subspecies with minimal bacteriostatic/bactericide concentrations [31,32,33]. Moreover, some of these AMPs have been successfully expressed in grapevines to control *Xf* in greenhouses [34,35,36]. Thus, AMPs are considered to be promising candidates for controlling this plant pathogen because of their antibacterial activity, low toxicity to the host plants, low propensity to incite bacteria-acquired resistance, and ability to be expressed in plants [37,38]. To search and explore whether there are more effective AMPs against *Xf* infection, this study has examined for the first time the efficiency of a set of nine potential peptides reported in the literature for their antimicrobial activity against Gram-negative and Gram-positive bacteria with biofilm activity [24,39], contrasting *Xf* in planta. All in vitro assays (antibiogram, OD, v-qPCR, biofilm inhibition, FM, and TEM) showed the high antimicrobial activity of the selected AMPs, among which GF19, FF13, and IL14 were found to be the most active at different concentrations. Most importantly, the selected AMPs showed supremacy in contrasting *Xf* infection in planta, demonstrating their prospects of being future antagonistic compounds for *Xf*. In addition, they had an innocuous impact on the eukaryotic plant and animal cells tested in this study, a finding that could trigger concrete AMP field experimental trials on *Xf*-infected trees.

Digging deeper into the efficiency of these AMPs, this study was able to distinguish between the biostatic and biocidal activities of the selected AMPs and their perfect ability to contrast biofilm formation. Furthermore, the efficiency of the selected AMPs against *Xf* at low concentrations was promising, representing an advantageous trait for their commercial production and application. N-acetylcysteine is a thiol compound that has been used as an antibacterial agent and inhibitor of biofilm formation which affects *Xf* strain 9a5c adhesion to glass surfaces, consequently reducing the biofilm biomass at 0, 2.0, and 6.0 mg/mL [18]; at 1 mg/mL, it was biocidal against *Xf* [18].

In general, several drawbacks limit the development of naturally occurring AMPs into useful phytopathogenic control agents [40], including stability issues, where the proteolytic degradation and the potential interaction of AMPs with enzymes might result in decreased antimicrobial activity [40]. Nanoparticle encapsulation systems have been extensively developed to enhance the bioavailability AMPs (such as natural polymerics [41,42], solid lipids [43], synthetic polymerics [44], liposomes [45], and inorganic nanoparticles [46]) in order to solve the limit of application of AMPs in plants.

High manufacturing costs are also another problem to be overcome. Researchers have been developing mimics or peptidomimetics with improved properties while maintaining the basic properties of membrane-active natural AMPs, for example, with amphipathic design and cationic charge. Multimeric (dendrimeric) peptides, protein epitope mimetics, lipidated peptides (synthetic), peptoids, oligoacyllysines, ceragenins, and other foldamers are some of the other approaches developed so far [47].

In this study, new bactericidal peptides, along with KL29 as previously described, have been identified, and their potential regarding new treatments for plant diseases caused by *Xylella fastidiosa* is realistic. Furthermore, these AMPs could be suitable candidates for environmentally and biologically friendly control measures in tandem with the preventive measures currently applied, contributing to greener agriculture, in view of European Union rules for quarantine organisms, especially for *Xf*. Furthermore, their ability to be expressed in plants makes them potentially useful in technologies through which transgenic plants can produce peptides to kill pathogens [48,49]. These AMPs need to be studied further in the future, covering a variety of combinations (e.g., mixtures of AMPs and combinations of AMPs and other antimicrobial agents). The effects of peptides on hormonal response, in gene expression in the preferred hosts of *Xf*, and in the field (whether alone or combined) should also be studied.

## Figures and Tables

**Table 1:** List of AMPs used for studying their effect on *Xylella fastidiosa*.

Peptide	Code	Sequence	Source	MIC (μM)			Reference
				*S. aureus*	*E. coli*	*X. fastidiosa*	
Ascaphin-8	GF19	GFKDLLKGAAKALVKTVLF-NH2	Frog	3.1	12.5	-	[25]
DASamP1	FF13	FFGKVLKLIRKIF-NH2	Synthetic	3.1	>100	-	[25]
DASamP2	IL14	IKWKKLLRAAKRIL-NH2	Synthetic	6.2	3.1	-	[25]
Lycotoxin I	IL25	IWLTALKFLGKHAAKHLAKQQLSKL	Spider	3.1	25	-	[25]
Maculatin 1.3	GF21	GLLGLLGSVVSHVVPAIVGHF-NH2	Frog	6.2	>100	-	[25]
Piscidin 1	FG22	FFHHIFRGIVHVGKTIHRLVTG	Fish	3.1	12.5	-	[25]
1036	VK13	VQFRIRVRIVIRK-NH2	Synthetic	-	-	12.5	[28]
RIJK2	RV12	RIVWVRIRRWFV-NH2	Synthetic	-	-	12.5	[21]
BP178	KL29	KKLFKKILKYL-AGPA-GIGKFLHSAK-KDEL-OH	Synthetic	-	-	12.5	[29]

**Table 2:** Effect of AMPs on growth inhibition of Xanthomonas albilineans.

AMP	Code	1 mM	150 μM	100 μM	50 μM	25 μM	12 μM	6 μM
Ascaphin-8	GF19	+++	+++	+++	+++	+++	+++	++
DASamP1	FF13	+++	+++	+++	+++	+++	+++	++
DASamP2	IL14	+++	+++	+++	+++	+++	+++	++
Lycotoxin I	IL25	+++	+++	+++	+++	++	-	-
Maculatin 1.3	GF21	+++	+++	+++	+++	++	-	-
Piscidin 1	FG22	+++	+++	+++	+++	++	-	-
1036	VK13	+++	+++	+++	+++	+++	++	+
RIJK2	RV12	+++	+++	+++	+++	+++	++	+
BP178	KL29	+++	+++	+++	+++	+++	+++	++

+++: Clear plaque formation; ++: turbid plaque formation; +: highly turbid plaque formation; -: no plaque formation.

## Data Availability

Not applicable.
